# Effects of Vegetables on Cardiovascular Diseases and Related Mechanisms

**DOI:** 10.3390/nu9080857

**Published:** 2017-08-10

**Authors:** Guo-Yi Tang, Xiao Meng, Ya Li, Cai-Ning Zhao, Qing Liu, Hua-Bin Li

**Affiliations:** 1 Guangdong Provincial Key Laboratory of Food, Nutrition and Health, Department of Nutrition, School of Public Health, Sun Yat-Sen University, Guangzhou 510080, China; tanggy5@mail2.sysu.edu.cn (G.-Y.T.); mengx7@mail2.sysu.edu.cn (X.M.); liya28@mail2.sysu.edu.cn (Y.L.); zhaocn@mail2.sysu.edu.cn (C.-N.Z.); liuq248@mail2.sysu.edu.cn (Q.L.); 2 South China Sea Bioresource Exploitation and Utilization Collaborative Innovation Center, Sun Yat-Sen University, Guangzhou 510006, China

**Keywords:** cardiovascular disease, vegetable, bioactive component, effect, mechanism

## Abstract

Epidemiological studies have shown that vegetable consumption is inversely related to the risk of cardiovascular diseases. Moreover, research has indicated that many vegetables like potatoes, soybeans, sesame, tomatoes, dioscorea, onions, celery, broccoli, lettuce and asparagus showed great potential in preventing and treating cardiovascular diseases, and vitamins, essential elements, dietary fibers, botanic proteins and phytochemicals were bioactive components. The cardioprotective effects of vegetables might involve antioxidation; anti-inflammation; anti-platelet; regulating blood pressure, blood glucose, and lipid profile; attenuating myocardial damage; and modulating relevant enzyme activities, gene expression, and signaling pathways as well as some other biomarkers associated to cardiovascular diseases. In addition, several vegetables and their bioactive components have been proven to protect against cardiovascular diseases in clinical trials. In this review, we analyze and summarize the effects of vegetables on cardiovascular diseases based on epidemiological studies, experimental research, and clinical trials, which are significant to the application of vegetables in prevention and treatment of cardiovascular diseases.

## 1. Introduction

Cardiovascular diseases (CVDs) have spread worldwide, and their prevalence is increasing dramatically [1,2]. CVDs have become one of the biggest threats to people’s health, as reported by the World Health Organization (WHO), and CVDs caused 17.7 million deaths (coronary heart disease (CHD): 7.4 million, stroke: 6.7 million) in 2015, accounting for 31% of all global deaths [3]. Besides high morbidity and mortality, CVDs also lead to serious disabilities and decrease the living standards of patients, creating a huge burden for individuals, families, and countries [4,5,6]. Therefore, it is urgent and worthwhile to investigate prevention and treatment strategies of CVDs [7].

CVDs are a class of chronic non-infectious diseases related to substantial complicated risk factors such as high blood pressure, hyperlipidemia, diabetes, overweight and obesity, metabolic syndrome, smoking, excessive alcohol consumption, imbalanced diet, and a lack of physical activity [6,8,9,10,11,12,13]. Efficient strategies can be applied to preventing and treating CVDs by targeting these risk factors, e.g., reducing blood pressure, regulating the blood lipid profile, reducing oxidative stress, modulating inflammatory status, inhibiting thrombosis, and attenuating myocardial damage as well as ameliorating metabolism syndrome [13,14,15,16,17,18,19]. Meanwhile, a healthy lifestyle, including a balanced diet, reasonable physical activity, moderate alcohol consumption, and stopping smoking, is beneficial to persons at high risk of CVDs [13,20,21,22,23]. Among these methods, establishing and insisting on a healthy eating pattern would be a substantial, sustainable, and economical choice. As recommended in the 2015–2020 Dietary Guidelines for Americans, people should follow a rational eating pattern with the aim of achieving and maintaining good health and lowering the risk of chronic diseases like CVDs, diabetes, overweight, and obesity throughout all stages of life [24].

It has been proven by epidemiological studies that vegetable consumption is negatively associated with the risks of CVDs [25,26,27,28,29,30]. Furthermore, evidence from experimental research suggested that many vegetables were effective in preventing CVDs, such as potatoes, soybeans, sesame, tomatoes, dioscorea, onions, celery, broccoli, lettuce, and asparagus [31,32,33,34,35]. Some bioactive components might account for the cardioprotective effects of vegetables, like vitamins, essential elements, dietary fibers, botanic proteins, and phytochemicals [36,37,38,39,40,41]. The potential mechanisms of action could involve antioxidation; anti-inflammation; anti-platelet; regulating blood glucose, lipid profile, and blood pressure; and attenuating myocardial damage [42,43,44,45]. In addition, clinical trials indicated that the consumption of several vegetables was beneficial to cardiovascular health [46,47,48,49]. The present review summarizes the effects of vegetables on CVD prevention and treatment, with special attention paid to the mechanisms of action.

## 2. Epidemiological Studies

Numerous epidemiological studies have indicated that vegetables were inversely associated with CVD incidence and many kinds of vegetables possessed cardioprotective effects, such as tomatoes, potatoes, onions, cereals, and cruciferous vegetables [26,27,28,29,30]. Moreover, a variety of bioactive components in vegetables have been proven to convey health benefits in preventing and treating CVDs, like botanical protein, dietary fiber, vitamins, essential elements, and phytochemicals [27,28,50].

### 2.1. Cross-Sectional Studies

Several cross-sectional studies evaluated the relationship between vegetable intake and CVD risks [26,51,52]. It was found that total cholesterol (TC), the TC/high density lipoprotein cholesterol (HDL-C) ratio, and hemoglobin A1c were significantly improved in women who consumed more than 10 servings/week of tomato-based food products compared to those consuming fewer than 1.5 servings/week [51]. Specifically, a significant improvement was observed in women with higher consumption compared to those with lower consumption, i.e., TC (5.38 mmol/L vs. 5.51 mmol/L, *p* = 0.029), the TC/HDL-C ratio (4.08 vs. 4.22, *p* = 0.046), and hemoglobin A1c (5.02% vs. 5.13%, *p* < 0.001), and consumers with higher intake were 31% (95% confidence intervals (CI): 6–50%), 40% (95% CI: 13–59%), and 66% (95% CI: 20–86%) less likely to have increased TC (≥6.21 mmol/L), low-density lipoprotein cholesterol (LDL-C) (≥4.14 mmol/L), and hemoglobin A1c (≥6%), respectively. In another study of 4774 Iranian subjects, significant correlations between potato intake and diabetes, high fasting blood sugar level as well as low serum HDL level were observed (odds ratio (OR): 1.38, 95% CI: 1.14–1.67, *p* < 0.001; OR: 1.40, 95% CI: 1.17–1.68, *p* < 0.001; OR: 1.10, 95% CI: 1.01–1.20, *p* = 0.02; respectively) [26]. These results suggested a potential effect of potato consumption on CVDs, as high fasting blood glucose, low serum HDL, and diabetes are recognized as CVD risk factors. Furthermore, it was indicated in a cross-sectional study with 3995 Mediterranean participants at high CVD risk that gazpacho (a Mediterranean vegetable-based cold soup that contains plenty of phytochemicals) intake was negatively correlated with hypertension [52]. It was found that both the systolic and diastolic blood pressure of the participants reduced with means of −1.9 mm Hg (95% CI: −3.4 to −0.6) and −2.6 mm Hg (95%CI: −4.2 to −1.0) and of −1.5 mm Hg (CI: −2.3 to −0.6) and −1.9 mm Hg (95% CI: −2.8 to −1.1), respectively, in moderate (1 to 19 g/day) and high (more than 20 g/day) gazpacho intake categories, compared with the control group. Moreover, the incidence of hypertension was decreased after gazpacho intake of 250 g/week and for high gazpacho intake groups compared with the control group, with OR = 0.85 (95% CI: 0.73–0.99) and OR = 0.73 (95% CI: 0.55–0.98), respectively.

### 2.2. Case-Control Studies

Similar results were illustrated in case-control studies investigating the association between vegetable intake and CVD risks [27,53,54,55]. In one study, the relationship between onion intake and acute myocardial infarction (MI) in Italy was analyzed [53]. Compared to the control group, the risks of acute MI for the group consuming less than one portion of onion per week and more than one portion per week significantly decreased (OR = 0.90, 95% CI: 0.69–1.21 and OR = 0.78, 95% CI: 0.56–0.99, respectively). Another case-control study in Korea suggested that consuming vegetables was inversely correlated with stroke risk [27]. It was shown that participants who consumed four to six servings of vegetables per day and more than six servings per day were 32% and 69% less likely, respectively, to suffer from stroke. Researchers also found that intake of vitamin B1, vitamin B2, vitamin B6, niacin, folate, calcium, and potassium was significantly inversely correlated with stroke risk. In addition, the effects of vegetable consumption on the connection between hypertension and the relative telomere length of peripheral leukocytes were measured in a study [54]. On one hand, it was reported that longer age-adjusted relative telomere length was related to higher vegetable consumption (*p* = 0.01). On the other hand, subjects with longer age-adjusted relative telomere length were 30% less likely to suffer from hypertension (OR = 0.70, 95% CI: 0.52–0.96, *p* = 0.03). The significant and negative connection between hypertension-relative telomere length and hypertension was only observed in those with greater (more than 150 g/day) vegetable intake (OR = 0.28, 95% CI: 0.14–0.57, *p* < 0.001), but not in those with lower (less than 50 g/day) vegetable intake (*p* = 0.008). Interestingly, in a study conducted in central Iran, evidence indicated that there was a marginally significant independent association between potato consumption and risk of stroke [55]. If compared with those with the lowest (5.3 ± 0.4 g/day) consumption, subjects with the highest (60.0 ± 6.1 g/day) potato consumption were more likely to have strokes (OR: 1.9, 95% CI: 1.0–3.6).

### 2.3. Cohort Studies

It has been confirmed by cohort studies that vegetable consumption was inversely relative to CVDs including hypertension, stroke, CHD, and even death [28,30,50]. Vegetables exerted a protective effect on CVD patients. A cohort study in Spain reported that cereal protein and fiber were negatively correlated with hypertension risk [28]. Cereal protein and fiber significantly lowered hypertension risk in participants in the highest quintile of intake compared with those in the lowest (hazard ratio (HR) = 0.5, 95% CI: 0.2–0.9, *p* = 0.06 and HR = 0.6, 95% CI 0.3–1.0, *p* = 0.05, respectively). Researchers also found that the risk reduction was more significant in older individuals compared to the young, in men compared to women, and in obese patients compared to those with a normal body weight. In another study, lycopene consumption was negatively correlated to CVD risks after a nine-year follow-up (HR = 0.83, 95% CI: 0.70–0.98), and to CHD risk after an 11-year follow-up (HR = 0.74, 95% CI: 0.58–0.94) [50]. Moreover, researchers found that vegetable intake could help decrease the risk of total mortality, with HRs (95% CIs) for total mortality across increasing quintiles of intake at 1 (reference), 0.88 (0.79–0.97), 0.88 (0.79–0.98), 0.76 (0.62–0.92), and 0.84 (0.69–1.00) for total vegetables (*p* = 0.03), and 1 (reference), 0.91 (0.84–0.98), 0.88 (0.77–1.00), 0.85 (0.76–0.96), and 0.78 (0.71–0.85) for cruciferous vegetables (*p* < 0.0001) [30]. Therefore, their findings supported the notion that increasing intake of vegetables, especially cruciferous vegetables, might help reduce CVD risk.

However, some studies found no significant inverse connection between vegetable consumption and CVD risks [56,57]. On the one hand, researchers focused on the connection between vegetable flavonoid intake and CVD risk in women in a cohort study, only to find that for both CVDs and important cardiovascular events, there was no significant linear trend across quintiles of vegetable flavonoid consumption (*p* = 0.63 and 0.80, respectively), and neither for the individual flavonol or flavone [56]. According to prospective data from another cohort study, broccoli consumption was not significantly associated with a reduction of CVD risk, and no significant association between broccoli flavonol or flavone consumption and nonfatal MI or fatal CHD risk was observed in U.S. women [57]. On the other hand, harmful components were found in a few kinds of vegetables or badly cooked ones [58,59]. The findings of a study indicated that habitually high consumption of soybean isoflavones might modestly but significantly increase the risks of ischemic stroke in women [58]. The HRs from the lowest (median intake: 6.0 mg/day) to the highest (median intake: 53.6 mg/day) quintiles were 1.00, 1.05, 1.10, 1.11, and 1.24, respectively (95% CI: 1.08–1.42, *p* = 0.002). In another study, researchers found that the HRs for subjects consuming four or more servings per week were 1.11 (95% CI: 0.96–1.28, *p* = 0.05) for baked, boiled, or mashed potatoes, 1.17 (95% CI: 1.07–1.27, *p* = 0.001) for French fries, and 0.97 (95% CI: 0.87–1.08, *p* = 0.98) for potato chips, compared with those consuming less than one serving per month [59]. These results indicated that a higher intake of badly cooked potato might increase the risks of developing hypertension independently and prospectively.

### 2.4. Other Epidemiological Studies

Besides the studies mentioned above, there are some epidemiological studies aiming to find an association between vegetable consumption and CVDs. Results from these epidemiological studies are also promising, as presented in Table 1.

In summary, evidence from most epidemiological studies suggested the significant negative relationship between vegetable consumption and the risks of CVDs. These vegetables specifically included tomato, potato, onion, cereal and cruciferous vegetables. Several kinds of components, like botanic protein, dietary fiber, vitamins (vitamin B1, vitamin B2, niacin and folate), essential elements (calcium and potassium) and phytochemicals (lycopene), might contribute to the cardioprotective effects of vegetables. However, in some studies, there were no observed significant inverse association between CVDs risks and intake of broccoli and vegetable flavonols. In some other studies, consuming potato, particularly badly cooked potato, could even increase the risk of CVD.

## 3. Experimental Research

### 3.1. Potatoes

People all over the word consume a large amount of potatoes per year. Potatoes have been found to benefit the cardiovascular system, thus they are worth investigating for the treatment and prevention of CVDs [42,43]. Researchers investigated the potential effects of a CA Mey (Hypoxidaceae) corm (African potato) aqueous extract (APE) on the cardiovascular system in experimental animal paradigms [42]. Firstly, APE (25–400 mg/mL) exhibited negative inotropic effects on guinea pig isolated electrically driven left atrial muscle preparations and negative chronotropic effects on spontaneously beating right ones, respectively, significantly (*p* < 0.05–0.001) and concentration-dependently. Secondly, APE concentration-dependently reduced or abolished the positive inotropic and chronotropic reactions of strips of atrial muscle from guinea pig induced by noradrenaline (1–100 μM) and calcium (Ca^2+^, 5–40 mM), which were not modified by exogenous administration of atropine (7.5 × 10^−7^ − 2.5 × 10^−6^ M) to the bath fluid. Thirdly, APE also caused a reduction or cessation of the rhythmic, spontaneous, myogenic contractions of portal veins in rats, significantly (*p* < 0.05–0.001) and concentration-dependently. Furthermore, APE reduced the systemic arterial blood pressure as well as heart rates of hypertensive rats, significantly (*p* < 0.05–0.001) and dose-dependently. Taken together, APE might be a natural candidate for cardiac dysfunction and essential hypertension remedy. In another study, cholesterol and triglyceride (TG) levels in plasma (−30%, *p* < 0.0001 and −36%, *p* < 0.05, respectively) and cholesterol levels in the liver (−42%, *p* < 0.0001) were significantly reduced in rats after three weeks of a potato-enriched diet [43]. Antioxidant status was also improved due to the intake of potato; additionally, thiobarbituric acid reactive substances (TBARS) levels in the heart were lowered and the vitamin E/TG (VE/TG) ratio in plasma was improved. These effects indicated that consumption of cooked potato could be a way of preventing CVDs. However, when researchers investigated the effects of soluble fiber extracted from potato pulp on risk factors for diabetes and CVDs in Goto–Kakizaki rats, no difference in hematological parameters was found; only the postprandial plasma TG concentration of rats was reduced, significantly but modestly [65]. These results might lead to a conclusion that plasma cholesterol or glycemic response could not be reduced by increased fermentation and production of propionate with diet-soluble fiber.

### 3.2. Soybeans

Soybeans are a common vegetable that can be used to extract oil and make soy milk. Polyphenols, mainly including phenolic acid and flavonoids like flavones and flavonols, are among the most important bioactive components extracted from soybeans. It was reported that phenolic acid mainly contributed to the antioxidant capacities of many natural products [66,67,68,69,70,71]. Many researchers suggested that polyphenols possessed biological effects like antioxidation and anti-inflammation, which in turn provided cardiovascular protection [37,44,45,72,73,74,75]. In an in vitro study, phenolic-rich extracts from soybeans were found to inhibit the activities of α-amylase, α-glucosidase, and angiotensin-I converting enzyme (ACE), which are key enzymes linked to diabetes and hypertension [44]. Thus, researchers came to the conclusion that soybeans have health-promoting effects including anti-diabetes and anti-hypertension. Another study investigated the effects of saponin (2-phenyl-benzopyrane), a soybean flavonoid, on glucose tolerance and risk factors for atherosclerosis [45]. In saponin-treated animals, the LDL-C/TG ratio was increased, and TG, very low-density lipoprotein cholesterol (VLDL-C), lipid hydroperoxide, and TC/HDL-C ratio were decreased. However, no effects were found on glucose tolerance, LDL-C, superoxide dismutase (SOD), and glutathione peroxidase (GPx) in the experimental groups. These observations indicated that saponin from soybeans might improve the serum lipid profile due to direct antioxidant activity.

It was also reported that soybeans contain considerable phytoestrogens, like isoflavones (mainly genistein and daidzein) and lignans, which are safe and natural estrogen receptor modulator alternatives to hormone therapy and possess antioxidant and cardioprotective effects [31,32,76]. Researchers analyzed the functional and anatomopathological effects of soybean extract and isoflavone on post-MI [76]. It was found that in the soybean extract group, a protective effect was observed 30 days after the MI. In another study, the cardioprotective effects of genistein from soybean extract on isoproterenol-treated H9c2 cardiomyoblast cells were investigated [31]. Results indicated that genistein administration could downregulate the expression of mitochondrial pro-apoptotic proteins such as Bad, caspase-3, caspase-8, and caspase-9 in H9c2 cells. Additionally, several survival proteins were expressed in H9c2 cells, including phosphor (p)-Akt, p-Bad, and p-Erk1/2. Moreover, researchers reported that genistein exerted cardioprotective effects partially due to the regulation of Erk1/2, Akt, and nuclear factor κ-light-chain-enhancer of activated B cells (NF-κB) proteins by inhibiting related pathways. It was also pointed out that genistein from soybeans not only reversed preexisting severe pulmonary hypertension but also prevented its progression into heart failure (HF) [32]. When genistein and daidzein were administered, significant neuroprotective effects and antioxidant activities were observed both in vitro and in vivo in ischemia/reperfusion (I/R)-like conditions [77]. Moreover, the effects of genistein from soybeans on blood pressure were evaluated in fructose-induced hypertensive rats [78]. Results showed that genistein administration could lower blood pressure and restore ACE, protein kinase C-β II, and nitric oxide (NO) synthase (NOS) expression.

Soybean protein is a well-known botanical protein that is regarded as a kind of complete protein, highly valuable in promoting health [79,80]. The cardioprotective effects of soybean protein have been proven by evaluating the association between dietary protein source, protein level, and serum lipid profile in male rats [79]. It was found that the total serum TG level was significantly lowered after long-term intake of soybean protein, indicating the possibility of reducing the risks of atherosclerosis. It was also reported that soybean protein possessed cardioprotective effects, partially by improving serum lipids via modifying the expression of sterol regulatory element-binding protein-2 and its downstream genes (hydroxymethylglutaryl-coenzyme A reductase and LDL receptor), and increasing the antioxidant activities of SOD and catalase [80].

It was reported that soybean products could be enhanced in nutritional value after fermentation [81]. For instance, doenjang was more effective at preventing diet-induced visceral fat accumulation than non-fermented soybeans in rats, by stimulating carnitine palmitoyltransferase-1 activity and suppressing fatty acid synthase activity, possibly due to the higher content of aglycone isoflavones [82]. Additionally, it was evaluated that regular intake of miso soup, a Japanese soybean paste, could alleviate salt-induced sympathoexcitation in mice with chronic pressure overload via inhibiting the hypothalamic MR–AT1R pathway [83]. Moreover, the effects of probiotic-fermented genetically modified (GM) soybean milk on hypercholesterolemia in hamsters were explored [84]. The observations suggested that serum total TG level decreased significantly (*p* < 0.05) after treatment with four kinds of soy milk (GM or non-GM; with or without probiotic fermentation), compared to the control group in a diet with high cholesterol. In addition, there was a significant difference between the GM and non-GM soy milk groups (*p* > 0.05) in total TG levels. Furthermore, the GM soy milk was found to reduce the risk of developing atherosclerosis by alleviating oxidative stress and diminishing atherosclerotic plaque formation in the aorta.

There are some other bioactive components in soybeans, such as unsaponifiables and oligosaccharides, which are beneficial to the cardiovascular system [85,86]. The protective effects of soybean unsaponifiables on the prefrontal cortex after global brain I/R injury in rats were investigated [85]. The results indicated that malondialdehyde (MDA) and tumor necrosis factor-α (TNF-α) levels, as well as the number of apoptotic neurons, were significantly decreased, while SOD activities were significantly increased, suggesting that soybean unsaponifiables had antioxidant and neuroprotective effects. In addition, the protective effects of soybean oligosaccharides on heart function against myocardium I/R injury were assessed in rats [86]. MDA level was upregulated, while antioxidant enzyme activities and the expression of p-JAK2 and p-STAT3 proteins were increased in the soybean-oligosaccharide-treated group. When rats were fed with soybean oligosaccharides, the cardiac contractile function was significantly recovered, the infarct size was reduced, and creatine kinase, aspartate transaminase, and lactate dehydrogenase activities were decreased as well.

### 3.3. Sesame

It has been demonstrated that extracts of sesame possessed strong antioxidant, anti-atherogenic, anti-thrombotic, and anti-hypertensive activity; thus, regularly consuming sesame whole grains or purified bioactive components would offer effective protection against CVDs [33,34,35]. In a study, chemical and biological model systems were used to access the free radical scavenging capacity and anti-atherogenic activity of *Sesamum indicum* seed extracts [33]. By Fe^3+^/ferricyanide complex and ferric reducing antioxidant power assays, it was reported that any dose (25–1000 μg/mL) of aqueous and ethanolic extracts significantly scavenged the NO, superoxide, 1-diphenyl-2-picrylhydrazyl, 2,2′-azinobis-(3-ethylbenzothiazoline-6-sulfonic acid)1, and hydroxyl radicals. In biological models, metal-induced lipid peroxidation in mitochondrial fractions, human serum, and LDL oxidation models was inhibited by both extracts. Moreover, in a lipoprotein kinetics study, the lag phase time was significantly (*p* < 0.05) increased by both extracts, while the oxidation rate as well as the conjugated dienes production was reduced. In another study, the anti-hypertensive effects of ACE inhibitory peptides from a sesame protein hydrolysate in spontaneously hypertensive rats (SHRs) were investigated [34]. The systolic blood pressure in SHRs was significantly and temporarily lowered by sesame peptide powder at 1 and 10 mg/kg, which might be due to ACE inhibitory activity. Moreover, the ACE activity was competitively inhibited by the representative peptides (Leu-Val-Tyr, Leu-Gln-Pro, and Leu-Lys-Tyr) isolated from sesame peptide powder at Ki = 0.92 μM, 0.50 μM, and 0.48 μM, respectively. According to the content ratio in sesame peptide powder, it was evident that a reconstituted sesame peptide mixture of Leu-Ser-Ala, Leu-Gln-Pro, Leu-Lys-Tyr, Ile-Val-Tyr, Val-Ile-Tyr, Leu-Val-Tyr, and Met-Leu-Pro-Ala-Tyr exhibited a strong anti-hypertensive effect on SHRs at doses of 3.63 and 36.3 μg/kg, which were responsible for more than 70% of the corresponding dosage for hypotensive effects induced by the sesame peptide powder. Furthermore, researchers focused on the anti-thrombotic effects of sesame, and found that Col/Chichibu/Maruteru-2/1995 and T016 varieties of sesame exhibited significant anti-thrombotic activity, while 00037803 was pro-thrombotic [35]. It was also observed that sesamol was the most effective component, followed by sesamolin and sesamin, which showed significant acute anti-thrombotic effects.

Although it was the fat-soluble constituents in the sesame that were thought to benefit the cardiovascular system, some studies demonstrated that defatted sesame seed extracts (DSSE) also possessed protective effects [87,88]. In a study, researchers evaluated the positive effects of DSSE using ischemia models [87]. It was found that DSSE (0.1–10 μg/mL) significantly blocked cell death and prevented lipid peroxidation induced by oxygen–glucose deprivation followed by reoxygenation. It was also evident that brain infarct volume was reduced in a dose-dependent manner, while sensory and motor function were improved by DSSE (30, 100, and 300 mg/kg, orally) administrated 0 h and 2 h after the onset of ischemia. Therefore, it could be concluded that DSSE might be effective in ischemia models due to the antioxidant activity. In another study, researchers investigated whether the neuroprotective effects of DSSE were related to brain edema [88]. The results showed that water content leakage was reduced by DSSE (30, 100, and 300 mg/kg, orally), but not Evans blue leakage. The Aquaporin 4 expression was inhibited by DSSE at 4 h but not at 24 h after ischemia. No effect on matrix metalloproteinase expressions and activities was observed. Herein, DSSE might be effective on brain edema due to the regulation of Aquaporin 4 during the acute phase of ischemia.

### 3.4. Tomatoes

Tomatoes were thought to have a considerable protective role in CVD; in particular, their bioactive component, lycopene, was found to exhibit significant antioxidant, anti-hypertensive, hypolipidemic, and anti-atherogenic effects in vivo and in vitro [36,39]. In a study, it was showed that the increase in serum MB-isoenzyme of creatine phosphokinase (CPK-MB) was prevented and cardiac cell injury was ameliorated by lycopene (1.7 and 3.5 mg/kg, intraparietally) and tomato extract (1.2 and 2.4 g/kg, intraparietally), respectively [36]. These results suggested that lycopene and tomato extract inhibited the cardiotoxicity induced by doxorubicin and could be used in combination with doxorubicin to alleviate the organ injury induced by free radicals. In another study, researchers investigated the effects of tomato extracts and carotenoids, like lycopene and lutein, on physiological function and NF-κB signaling in endothelial cells [39]. All carotenoids could cause a significant improvement in primary endothelial function, which was related to increase NO and decreased endothelin release. In addition, carotenoids effectively attenuated inflammatory NF-κB signaling, including reducing the adhesion of leukocytes induced by TNF-α, expression of adhesion molecules (AM) like inter-cellular adhesion molecule 1 (ICAM-1) and vascular cell adhesion molecule 1 (VCAM-1), nuclear translocation of NF-κB components, and reverting the inhibitor of κB ubiquitination. Additionally, carotenoids played a role in inhibiting NF-κB activation in transfected endothelial cells. Moreover, lutein combined with oleoresin synergistically precluded leukocytes’ adhesion.

Sapogenol, another major bioactive component in tomatoes, exhibited anti-atherogenic activities endowing tomatoes with cardioprotective effects [89,90]. It was reported that esculeogenin A, a new tomato sapogenol, ameliorated hyperlipidemia and atherosclerosis in ApoE-deficient mice via restraining cholesterol acyl-transferase [89]. Esculeogenin A markedly blocked the accumulation of cholesterol ester induced by acetylated LDL in human monocyte-derived macrophages and Chinese hamster ovary cells, dose-dependently. In addition, esculeogenin A prevented the expression of acyl-coenzyme A: cholesterol acyl-transferase (ACAT)-1 protein, and suppressed the activities of both ACAT-1 and ACAT-2. The levels of serum cholesterol, TG, LDL-C, as well as the proportion of atherosclerotic lesions in ApoE-deficient mice were significantly decreased by oral administration of esculeoside A, without any detectable side effects. In a similar study, tomatidine, a tomato sapogenol, was reported to significantly suppress the activity of cholesterol acyl-transferase and led to the reduction of atherogenesis [90].

In addition, the n-hexane extract of tomato exerted a protective effect against adrenaline-induced MI in rats [91]. The levels of MDA in heart and aspartate aminotransferase in serum were both significantly lowered in adrenaline-treated rats given a pre-treatment of tomato extract (1 mg/kg, 2 mg/kg) and vitamin E (50 mg/kg), which also significantly blocked myocardial necrosis. It could be concluded that the n-hexane extract of tomato possessed an antioxidative potential that might in turn prevent MI induced by catecholamine. Additionally, the anti-hypertensive effects of a tomato cultivar (DG03-9) rich in gamma-aminobutyric acid (GABA) were investigated in SHRs [92]. DG03-9 caused a significant reduction in systolic blood pressure with both single and chronic administration compared to the control. Moreover, researchers found that DG03-9 elicited a higher anti-hypertensive effect than the commonly consumed cultivar (Momotaro) did, and GABA exhibited a similar effect to DG03-9 in a comparable dose. Furthermore, it was reported that consuming cooked tomato sauce could preserve coronary endothelial function; improve HDL, apolipoprotein A-I, and apolipoprotein J protein profile; enhance endothelial NOS transcription and activation; and reduce DNA damage in the coronary arteries in dyslipidemic animals [93]. These bioactivities were responsible for the beneficial effects of cooked tomato sauce, i.e., lowing lipid peroxidation, increasing HDL antioxidant potential, and preventing diet-induced impairment of receptor-operated and non-receptor-operated endothelial-dependent coronary vasodilation.

### 3.5. Dioscorea

Dioscorea is a common vegetable widely used as traditional Chinese medicine, and contains a variety of bioactive components, like saponins, diosgenin, and flavonoids; it has been demonstrated that saponins have anti-thrombotic activity [40,41]. In a study, the total steroidal saponins derived from *Dioscorea zingiberensis* rhizomes blocked platelet aggregation and thrombosis dose-dependently, leading to prolonged activated partial thromboplastin time (APTT), thrombin time (TT), and prothrombin time (PT) in rats and prolonged bleeding time and clotting time in mice, suggesting ability to decrease CVD risk [40]. In another study, researchers evaluated the anti-thrombotic effects of four kinds of diosgenyl saponins [41]. The observations indicated that diosgenyl β-d-galactopyranosyl-(1→4)-β-d-glucopyranoside, a novel disaccharide saponin, exhibited outstanding efficiency in prolonging bleeding time. Moreover, it could significantly and dose-dependently block platelet aggregation, prolong APTT, and inhibit factor VIII activities in rats. Taken together, a conclusion could be drawn that diosgenyl β-d-galactopyranosyl-(1→4)-β-d-glucopyranoside had considerable anti-thrombotic activity. Moreover, the beneficial effects of total saponins exacted from three medicinal species of dioscorea, *Dioscorea nipponica* Makino, *Dioscorea panthaica* Prain et Burkill, and *Dioscorea zingiberensis*, against isoprenaline-induced myocardial ischemia were further investigated [94]. It was found that the total saponins from the three dioscorea significantly reduced activities of creatine kinase, lactate dehydrogenase, and aspartate aminotransferase; lowered the concentration of MDA; and increased activities of total SOD, catalase, GPx, and total antioxidant capacity, which was comparable between these three dioscorea. Additionally, heart tissue from total saponins groups revealed less severe histological damage. These results might partially explain why total saponins possess a cardioprotective efficacy for myocardial ischemia. Furthermore, saponins exhibited a potent neuroprotective property in attenuating severe injury induced by transient focal cerebral I/R, and the mechanism included anti-inflammatory and anti-apoptotic action [95]. It was reported that saponins markedly decreased neurological deficit scores, cerebral infarct volume, and brain edema in rats. Additionally, saponins increased neuron survival (Nissl bodies) and decreased caspase-3 in the hippocampal cornu Ammons 1 and cortex hemisphere of the ipsilateral ischemia. Moreover, pre-administration of saponins significantly decreased the inflammatory cytokines in serum induced by the middle cerebral artery occlusion, and markedly inhibited the downregulating anti-apoptotic Bcl-2 and upregulating proapoptotic Bax proteins.

It was reported that dioscorea, and its bioactive compound diosgenin in particular, exerted anti-thrombosis activity, possibly via promoting the anti-coagulation function and blocking platelet aggregation [96,97]. In a study, it was found that platelet aggregation, thrombosis and APTT, TT, and PT in rats were dose-dependently inhibited by diosgenin, while the bleeding time and clotting time were dose-dependently prolonged in mice [96]. It could be concluded that diosgenin extracted from *Dioscorea zingiberensis* possessed anti-thrombosis activities with a potential for CVD treatment. In another study, diosgenin was observed to alleviate cardiotoxicity induced by doxorubicin in mice [97]. In the heart tissue, diosgenin recovered the reduced activities of antioxidant enzymes, involving SOD and GPx. In addition, diosgenin significantly lowered the serum levels of cardiotoxicity markers, cardiac levels of TBARS and reactive oxygen species (ROS), caspase-3 activation, mitochondrial dysfunction, and the expression of NF-κB. Moreover, diosgenin increased the cardiac levels of cyclic guanosine monophosphate by modulating phosphodiesterase-5 activity and attenuating myocardial fibrosis. Meanwhile, it was confirmed that regulating protein kinase A and p38 could mediate the health benefits of diosgenin. These results implied that diosgenin possessed antioxidant and anti-apoptotic activities, as well as cyclic guanosine monophosphate modifying effects, which in turn protected the heart from cardiotoxicity induced by doxorubicin.

There are some other studies focusing on the beneficial effects of dioscorea on cardiovascular protection as well. More promisingly, other bioactive compounds contained in dioscorea have been identified, which might protect against MI and atherosclerosis [98,99]. In a study, results suggested that the flavonoid-rich portion of *Dioscorea bulbifera* Linn. could attenuate lipid peroxidation due to the capacity to scavenge free radicals and modulate energy-producing mitochondrial enzymes, suggesting a cardioprotective effect on isoproterenol-induced MI [98]. In another study, researchers arrived at the conclusion that an extract of Chinese yam, rich in β-sitosterol and ethyl linoleate, had the capability to prevent atherosclerosis, thus it could be a candidate for functional foods. It was reported that such extracts could inhibit the expression of inflammatory mediators, including TNF, NO, and inducible NOS, and the development of atherosclerotic lesions [99]. In addition, several studies also suggested the cardioprotective effects of dioscorea, of which the bioactive compounds might not have been identified [100,101]. In a study, it was confirmed that dioscorea rhizome exhibited antioxidative and anti-atherogenic effects on hyperlipidemic rabbits, suggesting that supplementation with dioscorea rhizome might be a possible way to reduce oxidative stress and attenuate atherosclerosis [100]. In another study, *Dioscorea opposita* Thunb. was found to exhibit anti-hypertensive effects on hypertensive rats through inhibiting the endothelin-converting enzymes as well as antioxidant activity [101]. After treatment, *Dioscorea opposita* Thunb. caused significant reductions in mean blood pressure, plasma endothelin and MDA concentration, plasma angiotensin-II activity, left ventricular hypertrophy, and cardiac mass index, while increasing the plasma SOD activity.

### 3.6. Onions

Onions are a commonly consumed vegetable all over the world, and contain bioactive components like phytochemicals. Onion extracts exhibited potent anti-atherogenic effects that were related to a variety of bioactivities [102,103]. In a study, onion (*Allium cepa* L.) extracts as well as the bioactive components quercetin and catechin were observed to enhance paraoxonase 1 activity and radical scavenging activity, which in turn prevented LDL oxidation and lipid peroxidation in male Wistar rats subjected to oxidative stress caused by mercuric chloride [102]. In another study, [103] onion extract was found to lessen atherosclerotic lesions, increase endogenous aortic hydrogen sulfide (H_2_S) production, and decrease plasma adrenomedulin content, aortic adrenomedulin content, aortic calcitonin receptor-like receptor, and receptor activity-modifying protein 1/2 mRNAs. Additionally, plasma GPx level, SOD activity, plasma endothelial NOS activity, and NO content were increased, while MDA and inflammatory response were reduced by onion extract. All of these effects made onions a potential candidate for anti-atherogenic therapy. 

Some experimental studies have suggested that onions have anti-thrombotic effects via platelet inhibitory response and inhibiting mitogen-activated protein kinase (MAPK) activation. Therefore, onion intake might have a capacity for preventing platelet-mediated CVDs [104,105,106]. In a study, results showed that onion could inhibit thrombosis induced by platelets in dogs [104]. It was demonstrated that periodic platelet-mediated thrombus formation followed by embolization caused a reduction in cyclic flow. However, in five dogs, 0.09 ± 0.01 mL/kg onion juice administered intravenously attenuated cyclic flow reductions within 20 min, followed by a 60 ± 14% (*p* = 0.002) reduction in collagen-induced ex vivo whole blood platelet aggregation. In addition, in six dogs given 2.0 g/kg onion homogenate intragastrically, cyclic flow reductions were lessened within 2.5–3 h in five of the dogs, accompanied by a 44 ± 24% (*p* = 0.04) reduction in ex vivo aggregation. Moreover, as measured by thrombosis/thrombolysis models in rodents in another study, a variety of onion cultivars exhibited natural anti-thrombotic effects [105]. First of all, researchers confirmed that Toyohira exerted marked anti-thrombotic activities as well as anti-platelet effects accompanied by thrombolytic activity. Meanwhile, Super Kita Momiji, 2935A, and K83211 exhibited only thrombolytic activity. Furthermore, researchers found no significant association between quercetin concentration and anti-thrombotic activity. Interestingly, the anti-thrombotic effects of quercetin-rich onion peel extracts (OPE) on arteries in rats were stated in another study [106]. The OPE markedly reduced blood TG and glucose without affecting blood cholesterol levels. In addition, in vivo arterial thrombosis was significantly abolished in groups fed with 2 mg and 10 mg OPE. Additionally, thrombin-induced expression of tissue factor in human umbilical vein endothelial cells, a coagulation initiator, was greatly diminished by the OPE. Furthermore, extracellular signal-regulated kinase (ESRK) and c-Jun N-terminal kinase (CJNK) signaling pathways activated by thrombin treatment were blocked by pre-treatment with OPE.

Onions were also found to have anti-hypertensive effects in some other experiments [107,108]. For instance, dietary onion decreased the TBARS in plasma in N(G)-nitro-l-arginine methyl ester (l-NAME)—induced-hypertensive rats and stroke-prone SHRs [107]. In addition, onions improved the nitrate/nitrite (products of NO) excreted in urine and the NOS activities in the kidneys in stroke-prone SHRs, but not in l-NAME- induced-hypertensive rats. These results might in part explain the mechanisms by which onion exerted an anti-hypertensive effect on these hypertensive rats. In addition, the anti-hypertensive effects of onion were observed with different mechanisms [108]. OPE was demonstrated to concentration-dependently reduce the aorta contractions induced by KCl or phenylephrine (*p* < 0.001). Moreover, the OPE activity could not be attenuated by removing aorta endothelium, or the inhibition of NO, cGMP and prostaglandin synthesis induced by l-NAME (100 μM), methylene blue (10 μM) and indomethacin (10 μM), respectively. In addition, the relaxation in phenylephrine-precontracted aorta mediated by OPE was not abolished by atropine, which blocked the acetylcholine-induced relaxation. Furthermore, after three weeks’ intervention with OPE, a reduction of blood pressure was observed in the hypertensive rats fed with fructose (*p* < 0.001).

### 3.7. Other Vegetables

Besides the vegetables investigated above, there are others that have beneficial effects on the cardiovascular system. Evidence from experimental research has suggested cardioprotective effects and mechanisms (Table 2).

**Figure 1:**
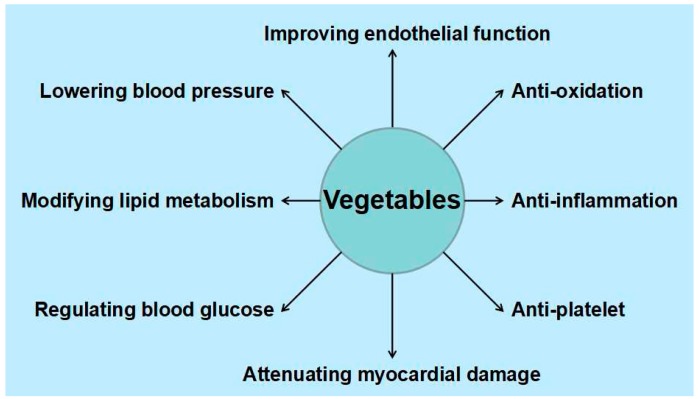
The cardioprotective effects of vegetables.

In summary, numerous experimental studies have indicated that vegetable consumption is potentially beneficial in preventing and treating CVD. As demonstrated, vegetables like potatoes, soybeans, sesame, tomatoes, dioscorea, and onions possess cardioprotective effects, for which a variety of bioactive components including vitamins, essential elements, dietary fiber, botanical proteins, and phytochemicals are responsible. In addition, the cardioprotective effects might include antioxidation, anti-inflammation, anti-platelet, lowering blood pressure, modifying lipid metabolism, regulating blood glucose, improving endothelial function, and attenuating myocardial damage (Figure 1). Moreover, the mechanisms of action might involve modulating related enzyme activity, gene expression, and signaling pathways as well as some other biomarkers associated with CVD risk (Table 3).

## 4. Clinical Trials

### 4.1. Whole Soybeans and Soy Milk

In addition to the findings in the abovementioned studies that soybeans possess a variety of bioactivities to prevent CVD, the results of many clinical trials have supported that soybean consumption might be a way to lower CVD risk and maintain cardiovascular health [46,47,48,49]. Soybeana had an effect on biomarkers of CVDs in elderly women with metabolic syndrome [46]. Compared to mean changes from baseline with the control group, LDL-C, VLDL-C, and apolipoprotein B100 levels were significantly improved in the whole soybean (35 g/day) intake group *(p* < 0.05), while fewer, significant improvements were observed in these variables in the textured soybean protein (35 g/day) group (*p* < 0.001). For apolipoprotein A-I, similar results were observed in the treatment groups (*p* < 0.01), in which serum TC was significantly decreased (*p* < 0.005). In all, soybeans and textured soybean protein could improve the lipid profile, but the former caused more considerable improvements than the latter. Moreover, consumption of soybean foods was found to improve the lipid profile in patients with hyperlipidemia [47]. According to the data measured in the separation of the group into equol producers (*n* = 30) and non-producers (*n* = 55), similar changes from baseline in LDL-C were observed (−9.3 ± 2.5% and −11.1 ± 1.6%, respectively, *p* = 0.834), with preservation of HDL-C and apolipoprotein A-I only in equol producers compared with changes in non-producers (HDL-C: +0.9 ± 2.7% compared with −4.3 ± 1.1%, *p* = 0.006; apolipoprotein A-I: −1.0 ± 1.1% compared with −4.7 ± 1.0%, *p* = 0.011). Moreover, soy milk consumption significantly reduced systolic blood pressure in type 2 diabetic patients with nephropathy when compared to cow’s milk consumption (percent change: −4.50% vs. 5.89%, *p* = 0.03) [48]. Additionally, intake of soy milk significantly reduced serum TG (percent change: −15.22% vs. 2.37%, *p* = 0.02), though these effects were not significant after adjustment for carbohydrate intake. Furthermore, data derived from another study indicated that the use of soybean products in comprehensive early rehabilitation therapy of patients with macrofocal MI significantly reduced the risk of arrhythmia [49].

### 4.2. Soybean Protein

Soybean protein could modestly improve the serum lipid profile as well as other risk factors related to CVD, as has been affirmed in a study involved 90 moderately hypercholesterolemic Chinese adults [138]. In a randomized controlled trial (RCT), soybean protein supplementation gave rise to a significant mean net change (95% CI) in plasma E-selectin of −3.93 ng/mL (−7.05 to −0.81 ng/mL, *p* = 0.014) compared with milk protein, and in plasma leptin of −2089.8 pg/mL (−3689.3 to −490.3 pg/mL, *p* = 0.011) compared with carbohydrate [139]. These observations indicated that soybean protein supplementation could reduce E-selectin and leptin levels in plasma. However, intake of either cow’s milk or a soy protein beverage for eight weeks did not alter soluble cell AM concentrations in pre-hypertensive or stage 1 hypertensive individuals, suggesting that neither beverage diminished atherosclerotic CVD risk in mildly hypertensive individuals by improving circulating cell AM concentrations [140].

### 4.3. Soybean Isoflavones

Soybean isoflavones, especially genistein and daidzein, are common phytoestrogens recognized as selective estrogen receptor modulators that possess cardioprotective effects in vitro and in vivo, but there is a lack of promising outcomes in clinical trials. In a six-month RCT, purified daidzein did not exhibit significant effects on body weight, body mass index, waist and hip circumferences, waist to hip ratio, body fat percentage, fat mass, and free fat mass in equol-producing postmenopausal women with prehypertension [141]. In the same study, it was found that purified daidzein had no significant effect on blood pressure and vascular function [142]. However, in the above two studies urinary isoflavones suggested good compliance of the patients with the interventions.

### 4.4. Combination of Soybean Isoflavones and Soybean Protein

Results from clinical trials also indicated that a combination of isoflavones and soybean protein might not be an effective intervention to prevent CVD [143,144]. In a RCT, isoflavone soybean protein (ISP) supplementation did not result in a significant reduction of subclinical atherosclerosis progression in postmenopausal women [143]. While subgroup analysis indicated that ISP supplementation could reduce subclinical atherosclerosis in healthy young women (median age: 53 years) less than five years postmenopausal who were at low risk for CVDs. In a double-blind randomized, placebo-controlled trial conducted among 180 postmenopausal Chinese women, soybean protein combined with isoflavones at the provided dosage (15 g soybean protein, 100 mg isoflavones) had no significant effect on measured cardiovascular risk factors, including serum HDL-C, LDL-C, TC, TG, and highly sensitive C-reactive protein [144].

### 4.5. Other Vegetables and Their Bioactive Components

Other vegetables and their bioactive components were also studied, such as sesame, tomatoes, broccoli, and onions, among which some were found to have properties promising for CVD prevention and treatment (Table 4).

In addition, healthy dietary patterns characterized by a high content of vegetables were important to reduce CVD risk [154,155]—for instance, the recommended Dietary Approach to Stop Hypertension (DASH) and the Mediterranean diet. The DASH diet suggests consumption of vegetables, fruits, and low-fat dairy products, and was found to result in a significant improvement of cardiovascular risk factors including BP, TC, and LDL, and cause a risk reduction for CVD incidence and mortality [156,157]. The Mediterranean dietary pattern is characterized by a high content of vegetables, as well as fruits and whole grains, and has been reported to decrease the incidence and mortality of CVDs, like CHD, MI, and stroke [158,159,160]. 

In summary, clinical trials showed that some specific vegetables had advantages in CVD prevention and treatment. Whole soybeans and their components (like soy protein) possessed potent cardioprotective effects. Moreover, some other vegetables, such as sesame, tomatoes, broccoli, and onions, were beneficial to CVD patients to some degree. On the other hand, no significant change in some biomarkers in subjects consuming tomato, broccoli, and soybean isoflavones was observed in some studies, so further clinical trials regarding the cardioprotective effects of vegetables are warranted. Furthermore, it is favorable to promote a healthy dietary pattern containing a high content of vegetable to reduce CVD risk.

## 5. Conclusions

The results from many epidemiological studies support the hypothesis that vegetable consumption is inversely correlated to the risk of CVDs. Moreover, numerous studies have suggested that many vegetables could be taken into consideration as candidates for CVD prevention and treatment, such as potatoes, soybeans, sesame, tomatoes, dioscorea, onions, celery, broccoli, lettuce, and asparagus, which contain varieties of bioactive components, including vitamins, essential elements, dietary fibers, botanical proteins, and phytochemicals. The cardioprotective effects of vegetables might include antioxidation, anti-inflammation, anti-platelet, lowering blood pressure, modifying lipid metabolism, regulating blood glucose, improving endothelial function, attenuating myocardial damage, modulating related enzyme activities, gene expressions and signaling pathways, as well as some other biomarkers associated with CVD risk. Furthermore, the cardioprotective effects of vegetables have also been observed in some clinical trials, though evidence was limited. Thus, consuming vegetables could help maintain cardiovascular health, and could be used as a substantial, sustainable, and economical strategy. In the future, more vegetables should be evaluated as to whether they have protective effects on the cardiovascular system, and bioactive components should be isolated and identified. Underlying mechanisms of action are also worth investigating. Meanwhile, more clinical trials should be conducted in this field.

## Figures and Tables

**Table 1:** Other vegetables associated with CVDs.

Vegetables	Subjects	Effects	References
Vegetables with carotenoids	U.S. male physicians aged 40–84 years (*n* = 15,220)	Lowered the risks of CHD	[60]
Onion quercetin		Incorporated into the atherosclerotic region, acted as a complementary antioxidant	[61]
soybean isoflavones	Chinese adults (*n* = 572)	Lowered serum TAG, carotid artery intima-media thickness, increased HDL-C	[62]
soybean foods, isoflavones	(Meta-analysis)	reduced ischemic heart disease, lowered blood LDL-C, improved endothelial function, slowed the progression of subclinical atherosclerosis	[63]
Green leafy vegetables	(Meta-analysis)	Reduced incidence of CVDs significantly (15.8%)	[29]
Nitrate-containing vegetables	Non-hypertensive subjects aged 20–70 years (*n* = 1546)	Had a protective effect against development of hypertension	[64]

**Table 2:** Other vegetables and their effects on CVDs.

Vegetables	Subjects	Effects and Mechanisms	References
*Apium graveolens* (seed)	Rats	Decreased blood pressure, increased heart rate	[109]
Celery (seed)	RAW264.7 macrophages	Lessened lipid droplets and TC content, decreased secretion of inflammatory cytokine TNF-α and interleukin (IL)-6, promoted cell viability, inhibited apoptosis, suppressed NF-κB, p65 and notch1 protein expressions	[110]
*Apium graveolens* (leaf)	Sprague Dawley rats	Decreased systolic blood pressure, cholesterol, TG, LDL and VLDL	[111]
*Asparagus officinalis*	SHRs	Lowered systolic blood pressure, urinary protein excretion/creatinine excretion ratio, creatinine clearance and ACE activity	[112]
Lettuce	Rats	Decreased LDL/HDL ratio and liver cholesterol levels, increased fecal total steroid excretion, depressed apparent absorption of dietary cholesterol, improved VE/TG ratio in plasma, limited lipid peroxidation in heart	[113]
Collard greens	SHRs	Modulated liver fatty acid composition, protected against elevations in atherogenic fatty acids	[114]
*Brassica oleracea* L.	In vitro thrombolytic model	Showed clot lysis activity	[115]
Rape (seed)	SHRs	Inhibited ACE, dilated mesenteric artery	[116]
Rape (seed)	SHRs	Inhibited ACE and renin activities, lowered blood pressure	[117]
Rape (seed)	SHRs	Reduced surface hydrophobicity, scavenged oxygen radicals, inhibited ACE, lowered blood pressure	[118]
Spinach	Balb/c mice	Decreased catalase, increased SOD activities, protected against doxorubicin-induced heart injury	[119]
Spinach	SHRs	Exerted anti-hypertensive activity	[120]
Spinach (leaf)	SHRs	Inhibited ACE, exerted anti-hypertensive activity	[121]
Pumpkin	In vitro	Antioxidant, inhibited α-glucosidase and ACE, anti-diabetic- and anti-hypertension	[122]
*Daucus carota*	In vitro, normotensive anesthetized rats	Lowered arterial blood pressure, inhibited spontaneously beating guinea pig atria and K^+^ -induced contractions of rabbit aorta	[123]
Lyophilized carrot	C57BL/6J mice	Increased total neutral sterols fecal excretion, increased antioxidant status and VE/TG ratio, lowered lipemia, regulated cholesterol metabolism	[124]
Carrot	In vitro, mice	Anti-thrombosis	[125]
Broccoli	stroke-prone SHRs	Attenuated oxidative stress, hypertension and inflammation	[126]
Broccoli	Rats	Protected mammalian hearts, activated survival proteins, improved post-ischemic ventricular function and pro-caspase 3 activities and redox cycling of thioredoxins, reduced myocardial infarct size, cardiomyocyte apoptosis and cytochrome c release	[127]
Broccoli	Rats	Protected against myocardial oxidative damage and cell death during I/R, inhibited markers of necrosis and apoptosis, decreased oxidative stress	[128]
Broccoli	Rats	Improved post-ischemic ventricular function, reduced MI and cardiomyocyte apoptosis	[129]
Corn	SHRs, in vitro	Inhibited ACE, lowered systolic blood pressure	[130]
Corn	In vitro	Antioxidant, inhibited a-glucosidase and ACE, anti-diabetic- and anti-hypertension	[122]
Purple corn	SHRs	Decreased blood pressure and heart rate	[131]
Maize	Wistar rats	Reduced infarct size, increased myocardial glutathione levels, modulated cardiac antioxidant defenses	[132]
Pea	Rats	Reduced MDA, tissue calcium concentration, myeloperoxidase and apoptosis indicator caspase-3, protected hearts from I/R injury	[133]
*Latyrus cicera*	Rats	Hindered free radical-mediated tissue injury, endothelial dysfunction and leukocyte recruitment, protected against splanchnic artery I/R-induced splanchnic injury	[134]
Pea	In vitro	Inhibited α-amylase and α-glucosidase and ACE	[135]
Pea	Weanling Han:SPRD-cy rats	Lowered serum creatinine and renal chemokine receptor 2 level	[136]
Pea	Rats	lowered plasma TC concentrations, affected cellular cholesterol homeostasis	[137]

**Table 3:** The mechanisms involved in the cardioprotective effects of vegetables.

Cardioprotective Effects	Mechanisms
Lower blood pressure	Inhibit ACE activity and hypothalamic MR-ATIR pathway, alleviate sympathoexcitation; improve protein kinase C-β II activity; modify relative telomere length of peripheral leucocyte, increase NOS expression; inhibit Ca^2+^ influx and K^+^ -induced contractions.
Regulate lipid metabolism	Decrease TC, TG, TAG, VLDL-C, TC/HDL-C ratio and atherosclerotic plaque formation, increase LDL-C/TG and VE/TG ratio; inhibit fatty acid synthase and ACAT activity, modulate energy producing mitochondrial enzymes; modify expression of ACAT and sterol regulatory element-binding protein-2 and its downstream genes.
Antioxidant	Scavenge free radicals (NO, superoxide, hydroxyl, TBARS); increase endogenous aortic H_2_S production; improve SOD, catalase, GPx, carnitine palmitoyltransferase-1 and paraoxonase 1 activity.
Improve endothelial function	Decrease endothelin and artery intima-media thickness, increase NO, improve apolipoprotein A-I and apolipoprotein J protein profile; inhibit endothelin-converting enzyme; diminish DNA damage.
Anti-inflammatory	Attenuate TNF-α induced leukocytes adhesion; reduce NF-κB, IL-6; inhibit expression of AM.
Anti-platelet	Prolong APTT, TT, PT, bleeding time and clotting time; inhibit MAPK, ESRK, factor VIII activities and c-Jun N-terminal kinase signaling pathways.
Attenuate myocardial damage	Decrease MDA, water content leakage and infarct size, increase cyclic guanosine monophosphate; inhibit creatine kinase, aspartate transaminase, lactate dehydrogenase and CPKMB activity, modulate protein kinase A, p38, and phosphodiesterase-5 activity; inhibit Bad, Bax, caspase-8, caspase-9, and caspase-3 and aquaporin 4 expression, increase phosphor (p)-Akt, p-Bad, p-Erk1/2, Bcl-2, p-JAK2 and p-STAT3.
Regulate blood glucose	Inhibit α-amylase and α-glucosidase activity; improve hemoglobin A1c and high fasting blood sugar level.

**Table 4:** Relationship between other vegetables and CVD.

Vegetables	Subjects	Effects	References
Sesame	Overweight or obese men and women (*n* = 33)	No improvement in markers of CVD risk	[145]
Tomato	Patients with grade-1 hypertension (*n* = 31)	Decreased blood pressure and TBARS level	[146]
Tomato	Healthy women (*n* = 18)	Improved serum antioxidant status, decreased vascular AM 1	[147]
Tomato	Healthy subjects (*n* = 40)	Decrease plasma TC, TG and several cellular and plasma inflammatory biomarkers, increase plasma HDL-C and IL-10	[148]
Tomato	Healthy middle-aged volunteers (*n* = 225)	No change in inflammatory markers, insulin resistance and sensitivity, lipid concentrations and arterial stiffness	[149]
Broccoli	Hypertensive individuals (*n* = 40)	No significant change in blood pressure and endothelial function measured by flow mediated dilation	[150]
Broccoli	Healthy Caucasian volunteers (*n* = 24)	Increased the urinary concentrations of sulforaphane metabolites and vitamin C, decreased the urinary concentrations of tetranor-PGEM, 11β-PGF2α and 11-dehydro-TXB2	[151]
Onion	Healthy men (*n* = 23)	Improved postprandial but not fasting flow-mediated vasodilation; did not alter systemic and forearm hemodynamics	[152]
Onion	Overweight-to-obese patients (*n* = 70)	Decreased 24 h, daytime and night-time systolic blood pressure in hypertensives; did not affect vasoactive biomarkers	[153]
